# Differences in Germination, Growth, and Fecundity Characteristics of Dicamba-Fluroxypyr-Resistant and Susceptible *Kochia scoparia*

**DOI:** 10.1371/journal.pone.0161533

**Published:** 2016-08-18

**Authors:** Vipan Kumar, Prashant Jha

**Affiliations:** Montana State University-Bozeman, Department of Research Centers, Southern Agricultural Research Center, Huntley, Montana, 59037, United States of America; Instituto Agricultura Sostenible, SPAIN

## Abstract

The widespread occurrence of herbicide-resistant (HR) *Kochia scoparia* is an increasing concern for growers in the US Great Plains and Canada. *K*. *scoparia* populations resistant to dicamba have been reported in six US states. Populations cross-resistant to dicamba and fluroxypyr have been reported from wheat fields in Montana, USA. It is unclear whether resistance to the auxinic herbicides (dicamba and/or fluroxypyr), can alter the fitness traits of *K*. *scoparia*. The objectives of this research were to compare the germination dynamics in response to thermal environment, vegetative growth and fecundity characteristics, and the relative competitive ability of dicamba-fluroxypyr–susceptible (S) vs.–resistant (R) *K*. *scoparia* selected from within a single segregating population (collected from wheat-fallow field in MT). S and R selected lines were developed after three generations of recurrent group selection. Compared to the S selected line, the R selected line had lower cumulative germination at all constant temperatures except 25°C, and at all alternating temperatures except 30/35°C. Also, the R selected line had delayed germination relative to the S selected line. The R had lower plant height, plant width, primary branches, total leaf area, stem diameter, and shoot dry weight compared with the S plants in the absence of competition. The reduction in seed production per plant resulted in a 39% fitness cost. The 1000-seed weight of R (1.6 g) was also less than that of S (2.6 g). When grown in an intraspecific competition at different mixture proportions, replacement series indices for the growth parameters further indicated that the R was less competitive than the S. Evident from this research, the dicamba-fluroxypyr–resistant R selected line is less likely to persist in a field population in the absence of the auxinic herbicides.

## Introduction

*Kochia scoparia* (L.) Schrad. (kochia) is one of the most troublesome broadleaf weed species in the US Great Plains, including Montana where it infests both croplands and noncroplands [1, 2]. This monecious, C4 summer annual plant exhibits several unique biological characteristics, including low seed dormancy, low seed persistence in the soil (< 2 yr), early seedling emergence, rapid growth, and tolerance to abiotic stresses [2–4]. *K*. *scoparia* is a highly competitive weed and can cause up to 95% yield reductions in agronomic crops [2]. It is a prolific seed producer (> 100,000 seeds plant^-1^), and exhibits a unique mechanism of seed dispersal through tumbling [5, 6]. Because of seed- and pollen-mediated gene flow coupled with protogynous flowering, this weed species exhibits genetic differentiation within and among populations [7–9].

Herbicide-resistant (HR) *K*. *scoparia* is an increasing concern for growers in the US Great Plains. *K*. *scoparia* resistant to photosystem II (PS II; Group 5 according to WSSA classification) and acetolactate synthase (ALS; Group 2) inhibitors have been reported across several states in this region [10]. More recently, *K*. *scoparia* with resistance to glyphosate (Group 9) have been reported in ten states in the US Great Plains, including Montana, and in southern Alberta, Saskatchewan, and Manitoba, Canada [10–13]. First reported in 1995 from Montana wheat fields, dicamba (Group 4)-resistant (DR) *K*. *scoparia* has been confirmed in six states in this region [10, 14].

We recently confirmed three *K*. *scoparia* populations with cross-resistance to dicamba (up to 6.8-folds) and fluroxypyr (up to 5.7-folds) from wheat-chemical fallow fields in north central Montana, USA [15]. The underlying mechanism(s) conferring resistance to auxinic herbicides (dicamba and fluroxypyr) in *K*. *scoparia* is not known with certainty. Previous studies have ruled out the possibility of differential absorption, translocation, or metabolism as a mechanism of resistance in *K*. *scoparia* and other auxinic-resistant weed species, including *Sinapis arvensis* L., *Galium spurium* L., *Centaurea solstitialis* L., *Stellaria media* (L.) Vill., reviewed in [16]. However, a reduced herbicide translocation and a higher rate of metabolism in roots conferred resistance in MCPA (2-methyl-4-chlorophenoxyacetic acid)-resistant *Galeopsis tetrahit* L. biotypes from Alberta, Canada [17]. It is speculated that mutations in genes encoding auxin-binding protein (ABP) may reduce the binding of auxinic herbicides, and confer resistance in *S*. *arvensis* [18]. A mutation in the auxin receptors affecting auxin-mediated responses, such as gravitropism and root growth inhibition, was hypothesized as a possible mechanism of resistance in DR *K*. *scoparia* biotypes from Montana [19]. Furthermore, differential gene expression studies had revealed up- and down-regulation of several transcripts following a dicamba treatment in those DR *K*. *scoparia* biotypes [20]. Nevertheless, the exact mechanism(s) of resistance to the auxinic herbicides in *K*. *scoparia* remains to be determined.

Natural mutations endowing herbicide resistance can confer fitness costs, either due to pleiotropic effects of the resistance alleles or linkage of the resistance alleles with one or more other loci that inhibit the normal plant metabolism, and divert energy and resources away from growth and reproduction [21–23]. Fitness cost endowed by herbicide resistance alleles is defined as the reduction in survival, growth, competitive ability, and reproductive success of an HR relative to a susceptible weed biotype, thereby reducing the frequency of the resistance alleles in a field population [22, 23]. A fitness cost endowed by auxinic-herbicide resistance was documented in MCPA-resistant *Ranunculus acris* L. [24]. It was reported that MCPA-resistant *R*. *acris* plants produced less biomass (g plant^-1^) compared with the susceptible plants, when grown in 1:1 mixtures under high densities in the absence of MCPA [24]. Similarly, auxinic-herbicide (MCPA, mecoprop, and dicamba)-resistant *S*. *arvensis* plants had reduced plant height, leaf area, root growth, and seed yield relative to susceptible plants [25, 26]. In contrast, no difference in shoot biomass of a *K*. *scoparia* biotype cross-resistant to dicamba and fluroxypyr (HRdf) relative to a susceptible biotype from Montana was observed when grown in competition with wheat under varying nitrogen levels [27]; however, in the previous study, differences in seed germination, vegetative growth parameters other than shoot biomass, and fecundity characteristics between the two biotypes were not quantified. Further, no definite conclusions on the fitness endowed by the auxinic-herbicide resistance alleles in *K*. *scoparia* could be drawn from the previous study [27], because the HRdf and susceptible biotypes were collected from two different geographical locations (different genetic backgrounds), with a different cropping and herbicide-use history [22]. It is important to ensure that HR and susceptible individuals used in fitness-cost studies share a common genetic background [22, 23].

The objectives of the research were to investigate (1) seed germination pattern of dicamba-fluroxypyr–resistant vs.–susceptible *K*. *scoparia* under different constant and alternating temperature regimes, (2) relative growth and fecundity characteristics of dicamba-fluroxypyr–resistant and –susceptible *K*. *scoparia* in the absence of competition, and (3) competitive ability of dicamba-fluroxypyr–resistant relative to susceptible *K*. *scoparia* in replacement series experiments in the greenhouse.

## Materials and Methods

### Plant material and genetic background control

*K*. *scoparia* seeds were collected in fall 2011 from a confirmed dicamba-fluroxypyr–resistant segregating population in a wheat field (wheat-chemical fallow rotation) in the Choteau County, Montana, USA [15]. The field had a history of more than 12 yr under no-tillage wheat-fallow rotation, with the repeated use of dicamba and/or fluroxypyr for weed control in wheat, and dicamba in conjunction with 2,4-D and glyphosate for weed control in chemical fallow. The grower/land owner provided the permission to enter the field for weed seed collection. We acknowledge that the seed sampling did not involve any endangered or protected species.

Field-collected seeds were sown on the surface of 53 by 35 by 10 cm flats filled with a commercial potting media (VermiSoil^™^, Vermicrop Organics, 4265 Duluth Ave, Rocklin, CA) in a greenhouse at the Montana State University Southern Agricultural Research Center, near Huntley, Montana. The greenhouse was maintained at 26/23 ± 3°C day/night temperatures and 16/8 h day/night photoperiods, and the supplemental photoperiod was obtained with metal halide lamps (400 μmol m^-2^ s^-1^). Emerged seedlings were transplanted into 10-L plastic pots. A total of 100 plants were grown for 6 wk, and two sets of vegetative clones (four shoot cuttings per set) from every plant were prepared as described in [7]. At the 10 cm height, one set of clones from each plant was treated with 0, 1/2, 1, and 2X where X = field-use rate of 280 g ae ha^-1^ of dicamba (Banvel^®^, BASF Corporation, Research Triangle Park, NC), and the other set of clones from the same plant were treated with 0, 1/2, 1, and 2X where X = field-use rate of 140 g ae ha^-1^ of fluroxypyr (Starane^®^ Ultra, Dow AgroSciences LLC., 9330 Zionsville Road, Indianapolis, IN). Applications were made inside a stationary cabinet spray chamber (Research Track Sprayer, De Vries Manufacturing, RR 1 Box 184, Hollandale, MN) equipped with an even flat-fan nozzle tip (TeeJet 8001EXR, Spraying System Co., Wheaton, IL), calibrated to deliver 94 L ha^-1^ of spray solution at 276 kPa. After the herbicide application, clones were returned to the greenhouse, watered as needed to avoid moisture stress, and fertilized [Miracle-Gro water soluble fertilizer (24-8-16), Scotts Miracle-Gro Products Inc., 14111Scottslawn Road, Marysville, OH] weekly to maintain good plant growth. At 28 DAT, clones that did not survive 1/2X or higher doses of dicamba or fluroxypyr were characterized as ‘dicamba-fluroxypyr–susceptible’. Clones that survived 2X rate of dicamba or fluroxypyr were characterized as ‘dicamba-fluroxypyr–resistant’. Those susceptible and resistant clones were used for developing the selected lines, referred to as S and R, respectively.

Twenty clones each of R and S were grown separately, and a group of cloned plants were subjected to restricted cross-pollination to prevent gene flow between the resistant and susceptible phenotypes. Upon maturity, seeds were collected, and bulked separately for R and S. Selected lines were developed after three generations of group (to avoid inbreeding depression) recurrent selection (two generations with dicamba or fluroxypyr, and one generation without the herbicides to obtain sufficient amount of seeds for the experiments). This methodology enabled the development of selected lines from discrete dicamba-fluroxypyr–resistant and –susceptible plants derived from within a single *K*. *scoparia* population in the field; hence, assuming a common genetic background [22, 23]. The levels of resistance of R selected line to dicamba and fluroxypyr were 6.8-folds and 5.7-folds, respectively, determined in a separate dose-response study [15].

### Seed germination and viability

Germination of seeds obtained from S and R *K*. *scoparia* selected lines was evaluated at constant (5, 10, 15, 20, 25, 30, 35 C) and alternating temperatures (5/10, 10/15, 15/20, 20/25, 25/30, 30/35 C, 12 h/12 h cycle). Two separate experiments (one for constant and other for alternating temperatures) were conducted at the Montana State University Southern Agricultural Research Center (MSU-SARC) near Huntley, MT in the fall 2013, in a completely randomized design with six replications. Experiments were repeated in time. Seeds of both *K*. *scoparia* selected lines were surface-sterilized by using 1% w/v sodium hypochlorite solution for 1 min, and then rinsed with double-distilled water twice before initiating the germination experiments. All experiments were conducted on freshly harvested, fully-matured seeds of *K*. *scoparia* selected lines.

Fifty seeds were evenly placed between two layers of filter papers (Whatman^®^, Grade 2, Sigma-Aldrich Inc., St. Louis, MO 63178, USA) moistened with 10 ml of distilled water in a 10-cm-diam petri dish (Sigma-Aldrich). Light is not required for germination of *K*. *scoparia* seed [28]. Therefore, petri dishes were wrapped with aluminum foil, and placed in incubators (VMR International, Sheldon Manufacturing Inc., Cornelius, OR 97113, USA) set at either constant or alternating temperatures. Seeds with visible and uncoiled tip of radicles were considered as germinated [29]. The numbers of germinated seeds were counted daily. No further germination occurred after 15 d of incubation in both runs, and germination counts were terminated at the end of 28 d. Subsequently, non-germinated seeds were tested for viability using a 1% (w/v) tetrazolium chloride solution [30].

### Noncompetitive growth and seed production

Greenhouse experiments were conducted at the MSU-SARC near Huntley, MT in the fall 2013 (run 1) to determine the vegetative growth and fecundity characteristics of dicamba-fluroxypyr–resistant (R) and *–*susceptible (S) selected lines of *K*. *scoparia* in the absence of competition, and repeated in fall 2014 (run 2). Seeds of S and R *K*. *scoparia* were separately sown on germination flats containing the commercial potting media in the greenhouse as previously described. The emerged *K*. *scoparia* seedlings (2- to 3-cm tall) were transplanted into 20-L plastic pots containing the same potting media. During pot filling, slow release granular fertilizer (Osmocote Vegetable and Bedding [14-14-14], Scotts Company LLC, Marysville, OH) at the rate of 5 g L^-1^ was added to the potting media. *K*. *scoparia* plants were watered as needed, and fertilized biweekly to maintain good growth. Pots were evenly spaced (36 cm apart) in the greenhouse to ensure adequate room for growth of each plant and avoid any interplant shading; pots were re-randomized every week.

The study was conducted in a completely randomized design, with six replications in each run, repeated in time. Treatments included two *K*. *scoparia* selected lines, i.e., S and R. In each experimental run, there were total of 132 pots (2 *K*. *scoparia* selected lines × 11 harvest timings × 6 replications). The destructive harvesting of *K*. *scoparia* plants was initiated at two wk after transplanting (WAT), and terminated at the beginning of flowering. There were a total of ten harvest dates at weekly interval for assessing vegetative growth parameters and one end-season harvest date for assessing reproductive parameters. At each harvest date, six *K*. *scoparia* plants per selected line were cut at the soil surface, and the vegetative traits including, plant height (measured from the base of the plant to the terminal leaf), plant width (maximum canopy diameter), number of primary branches, stem diameter, and total leaf area were measured. Stem diameter was measured using 15-cm digital caliper (Pittsburgh^®^, Harbor Freight Tools Co., 3491 Mission Oaks Blvd., PO Box 6009, Camarillo, CA). Leaves from each plant were manually separated from the branches and stem, and the total leaf area plant^-1^ was measured using a leaf area meter (LI-COR 3000, LII-COR, Inc., Lincoln, NE). The vegetative biomass (leaves, stems/branches) was oven dried at 70°C for 72 h, and the shoot dry weight plant^-1^ was determined.

For reproductive parameters, six plants of each *K*. *scoparia* selected line were retained. Individual plants were covered with pollination bags (DelStar Technologies, Inc., 601 Industrial Drive, Middletown, DE) to prevent cross pollination. The number of days required to reach flower initiation stage was recorded. At maturity, plants were manually harvested, and stored in separate paper bags. After separation from the inflorescence, seeds of each *K*. *scoparia* plant were air dried at room temperature, and cleaned using a coarse-mesh (2-mm) screen. The small chaff and other debris were removed from the seed sample by using an air-propelled column blower (Seedburo Equipment Co., 2293 S. Mt. Prospect Road, Des Plaines, IL). The 1000-seed mass and the total seed mass plant^-1^ were recorded to estimate the seed production plant^-1^.

### Competitive growth study

Greenhouse experiments were conducted at the MSU-SARC near Huntley, MT during fall 2013 (run 1), and repeated in spring 2014 (run 2). Replacement series experiments were conducted to determine the relative fitness of S vs. R *K*. *scoparia* selected lines. Experiments were conducted at MSU-SARC near Huntley, in a completely randomized design (CRD) with five replications. Treatments included five different mixture proportions (intraspecific densities) of S vs. R (100:0, 75:25, 50:50, 25:75, and 0:100). Seedlings of each *K*. *scoparia* selected line were separately grown in plastic trays as previously described. After emergence (2- to 3-cm tall), seedlings were transplanted into 20-L plastic pots containing a commercial potting media fertilized with 5 g L^-1^ of Osmocote. Seedlings were transplanted in a grid pattern (equidistant from the edge of the pot) at a density of four plants pot^-1^ (representing a density of 84 plants m^-2^), for each mixture proportion. Pots were watered as needed, and fertilized on a bi-weekly basis to maintain good growth.

Pots were re-randomized on a weekly basis to avoid any variation associated with the light regime on the greenhouse bench. At 8 WAT, *K*. *scoparia* plants were individually cut at the soil surface, and plant height, plant width, primary branches, stem diameter, and total leaf area plant^-1^ were recorded. Plants were oven dried at 70°C for 72 h, and the shoot dry weight plant^-1^ was determined. Since the authors work at the MSU-SARC, Huntley, Montana, no permission was needed to conduct the greenhouse and laboratory experiments.

### Statistical analyses

The cumulative seed germination (%) of selected lines S and R was determined for each temperature treatment, and regressed against the time of incubation (h) using a three-parameter log-logistic model [31, 32]:

y = {d /1 + exp [b (log t−log e)]} (1)

Where *y* refers to cumulative germination (%), *d* is the upper limit of the curve, *b* is the slope, *e* is the incubation time (h) required for 50% seed germination referred as I_50_, and *t* is the incubation time. Parameter estimates, standard errors, I_50_ and I_90_ values (incubation time required for 50 and 90% cumulative seed germination, respectively) of S and R *K*. *scoparia* at each temperature treatment were determined using the *drc* package in ‘*R’* software [32]. Parameter estimates of S and R selected lines at each temperature treatment were compared using the ‘*compParm*’ statement in the *drc* package.

For the noncompetitive growth and seed production study, all data were subjected to ANOVA using PROC MIXED in SAS to test the significance of experimental run, *K*. *scoparia* selected line, WAT, and their interactions. Data on vegetative growth attributes were analyzed for each harvest date separately. The ANOVA assumptions were tested using PROC UNIVARIATE and PROC GLM in SAS, and all the data met both the assumptions (normality of residuals and homogeneity of variance). Means were separated using Fisher's protected LSD test at P < 0.05.

In the competitive growth study, data collected on vegetative growth parameters of S and R selected lines were subjected to ANOVA using PROC MIXED in SAS. To measure the relative competitiveness between S and R, replacement series indices were estimated [33–35]:

1. Relative yield (RY):
  RY (S) = P (Smix/Smono) (2)
  RY (R) = (1−P) (Rmix/Rmono) (3)
  where RY (S) and RY (R) are relative yields of the S and R *K*. *scoparia* selected lines, respectively; *P* is the proportion of the S or R in the mixture; S_mix_ and R_mix_ are yields of S and R in the mixture, respectively; S_mono_ and R_mono_ are yields of S and R in monoculture, respectively.
2. Competitive ratio (CR):
  CR = [(1−P)/P] [RY (S)/RY (R)] (4)
  The CR values for each mixture proportion were compared with the expected values (H_0_ = 1.0) using a one-sample *t* test (α = 0.05).
3. Relative crowding coefficient (RCC):
  RCC (S) = [(1−P)/P] [RY (S)/ (1−RY (S))] (5)
  RCC (R) = [(1−P)/P] [RY (R)/ (1−RY (R))] (6)
  The difference between RCC (S) and RCC (R) was tested using student’s *t* test (α = 0.05).
4. Aggressiveness index (AI):
  AI = (RY (S)/2P)−{RY (R)/[2(1−P)]} (7)

The estimated AI value for a growth parameter was tested against the expected value (H_0_ = 0) using a one-sample *t* test (α = 0.05). Replacement series indices including, CR, RCC (S), RCC (R), and AI indices were only computed for the equal mixture proportion (50:50) of S vs. R *K*. *scoparia*.

## Results

### Seed germination

The experimental run-by-treatment interaction was not significant (P = 0.231); therefore, data from the two runs were pooled. Lack-of-fit tests indicated that the nonlinear models accurately described the data (P > 0.05). More than 95% seeds of both *K*. *scoparia* selected lines were viable at all temperatures tested in both runs. The differences in cumulative germination between S and R selected lines were evident at all constant temperatures tested, except at 25°C. The R had up to 40% less cumulative germination (*d* parameter estimate) compared with the S selected line at constant temperatures of 5, 10, 15, 20, 30 and 35°C (Table 1). Based on the fitted three-parameter log-logistic models, the I_50_ and I_90_ values (incubation times required to achieve 50 and 90% cumulative seed germination, respectively) of S and R differed at majority of the constant temperatures tested. Seeds of the S *K*. *scoparia* germinated 12 to 66 h earlier than the R to achieve 50% cumulative germination at 10, 15, 30, and 35°C. Furthermore, seeds of S selected line germinated 61 to 145 h sooner than the R selected line to achieve 90% cumulative germination (I_90_ values) across all constant temperature tested, except 20°C (Table 1).

**Table 1:** Regression parameters estimated from the three-parameter log-logistic model (Eq 1: *d* = upper limit, *b* = slope) for percent cumulative germination of dicamba-fluroxypyr–susceptible (S) and –resistant (R) *K*. *scoparia* at various constant temperatures averaged over experimental runs.

Temperature (°C)		Regression parameter estimates			
	Selected line^a^	*d* (± SE)^b^	*b* (± SE)	I_50_ (95% CI)^c^^,^ ^d^	I_90_ (95% CI)^c^^,^ ^d^
**5**	S	89.2 (1.2) a	- 4.3 (0.4)	88 (84–92) a	146 (130–162) b
	R	53.2 (1.9) b	- 2.7 (0.3)	93 (83–103) a	210 (170–250) a
**10**	S	87.3 (2.5) a	- 1.9 (0.1)	69 (62–76) b	181 (134–228) b
	R	47.0 (1.2) b	-2.3 (0.3)	100 (91–109) a	318 (244–392) a
**15**	S	91.8 (0.9) a	-2.2 (0.2)	32 (29–35) b	186 (170–202) b
	R	75.6 (1.4) b	-0.9 (0.1)	98 (50–146) a	331 (210–452) a
**20**	S	108.3 (3.4) a	- 1.4 (0.1)	55 (47–63) a	252 (155–349) a
	R	90.0 (3.7) b	1.4 (0.2)	58 (49–67) a	276 (145–407) a
**25**	S	100.9 (1.2) a	- 2.0 (0.1)	39 (36–42) a	113 (94–132) b
	R	94.8 (2.6) a	- 1.4 (0.2)	43 (38–48) a	201 (175–227) a
**30**	S	93.8 (1.2) a	-2.4 (0.4)	24 (21–27) b	58 (42–74) b
	R	78.3 (3.0) b	-1.4 (0.2)	36 (30–42) a	169 (90–248) a
**35**	S	97.6 (1.4) a	-1.6 (0.2)	23 (20–26) b	87 (60–114) b
	R	71.3 (2.1) b	-1.5 (0.2)	35 (30–40) a	148 (138–158) a

^a^ S, dicamba-fluroxypyr–susceptible *K*. *scoparia* selected line; R, dicamba-fluroxypyr–resistant *K*. *scoparia* selected line; SE, standard error of mean; CI, confidence interval.

^b^ Value in parenthesis represent standard error of the mean.

^c^ I_50_ is the time (h) of incubation required for 50% cumulative seed germination; I_90_ is the time (h) of incubation required for 90% cumulative seed germination.

^d^ Within each temperature, *d*, I_50_, or I_90_ values followed by the same letter are not significantly different based on the approximate *t* test using the “*CompParm*” command in the drc package, *R* software [32].

The R selected line had up to 32% less cumulative germination (*d* parameter estimate) compared with the S selected line at all alternating temperatures tested, except at 30/35°C (Table 2). At alternating temperatures of 5/10 and 10/15°C, seeds of S germinated 14 to 32 h earlier than R to achieve 50% cumulative germination (I_50_ values) (Table 2). Similarly, the S germinated 17 to 45 h sooner than the R selected line to achieve 90% cumulative germination (I_90_ values) at alternating temperatures of 15/20 and 25/30°C. No difference in I_50_ or I_90_ values was evident at 30/35°C alternating temperature.

**Table 2:** Regression parameters estimated from the three-parameter log-logistic model (Eq 1: *d* = upper limit, *b* = slope) for percent cumulative germination of dicamba-fluroxypyr–susceptible (S) and –resistant (R) *K*. *scoparia* at various alternating (12/12 h cycle) temperatures averaged over experimental runs.

Temperature (°C)		Regression parameters estimates			
	Selected line^a^	*d* (± SE)^b^	*b* (± SE)	I_50_ (95% CI)^c^^,^ ^d^	I_90_ (95% CI)^c^^,^ ^d^
**5/10**	S	88.1 (3.9) a	- 1.4 (0.1)	74 (65–83) b	367 (302–432) a
	R	65.4 (2.3) b	-1.5 (0.1)	106 (90–122) a	392 (350–434) a
**10/15**	S	95.6 (2.5) a	-1.4 (0,1)	56 (49–63) b	278 (178–378) a
	R	68.8 (2.0) b	-1.3 (0.2)	70 (63–77) a	312 (275–349) a
**15/20**	S	93.9 (0.9) a	-2.1 (0.1)	34 (30–38) a	97 (82–112) b
	R	74.5 (1.6) b	-1.5 (0.2)	35 (31–39) a	142 (121–163) a
**20/25**	S	95.7 (0.6) a	-2.0 (0.1)	31 (27–35) a	102 (92–112) a
	R	63.9 (0.6) b	-1.8 (0.1)	35 (30–40) a	102 (84–120) a
**25/30**	S	96.8 (1.0) a	-2.8 (0.4)	26 (21–31) a	57 (48–66) b
	R	85.2 (1.2) b	-2.3 (0.3)	29 (25–33) a	74 (68–80) a
**30/35**	S	95.3 (0.8) a	-2.3 (0.2)	30 (26–34) a	75 (64–86) a
	R	92.4 (0.8) a	-2.1 (0.1)	32 (29–35) a	81 (74–89) a

^a^ S, dicamba-fluroxypyr–susceptible selected line; R, dicamba-fluroxypyr–resistant selected line; SE, standard error of mean; CI, confidence interval.

^b^ Value in parenthesis represent standard error of the mean.

^c^ I_50_ is the time (h) of incubation required for 50% cumulative seed germination; I_90_ is the time (h) of incubation required for 90% cumulative seed germination.

^d^ Within each temperature, *d*, I_50_, or I_90_ values followed by the same letter are not significantly different based on the approximate *t* test using the “*CompParm*” command in the drc package, *R* software [32].

### Noncompetitive growth and seed production

**Fig 1:**
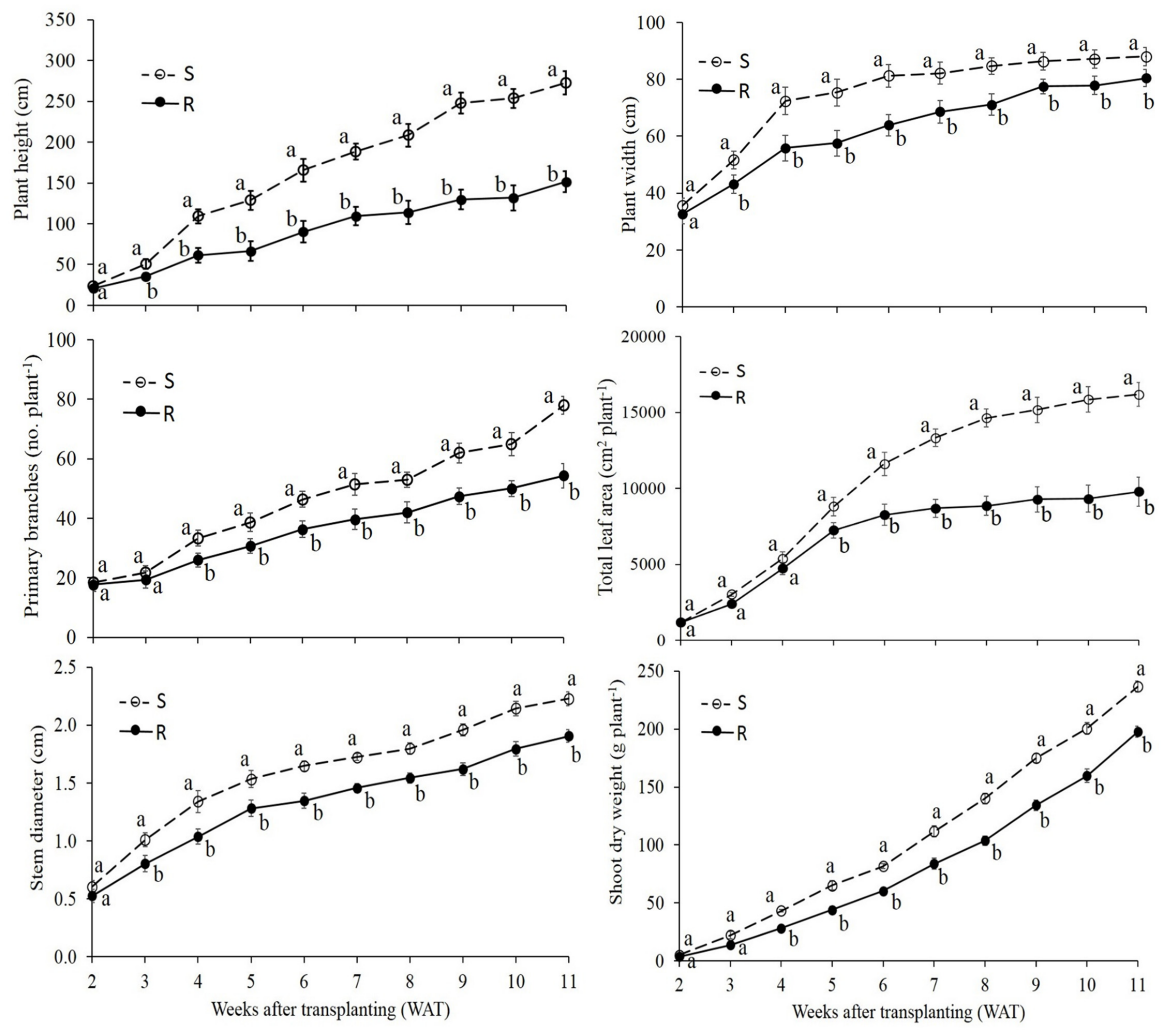
Vegetative growth parameters of dicamba-fluroxypyr–susceptible (S) and –resistant (R) *K*. *scoparia* in the noncompetitive growth study averaged over experimental runs. Vertical bar denotes the standard error of the mean. For a growth parameter, similar letters at a harvest date, indicate no difference between the means of R and S selected lines based on the Fisher’s protected LSD test at P < 0.05.

The experimental run-by-treatment interaction for all the vegetative or reproductive growth parameters was nonsignificant; therefore, all data were pooled across runs (α = 0.05). Results from the ANOVA indicated that the vegetative growth parameters of the S were significantly different than R selected line at a majority of harvest dates (Fig 1). The R had up to 44% less plant height, 24% less plant width, 31% less primary branches, 41% less total leaf area, 22% less stem diameter, and 39% less aboveground shoot dry weight compared with the S *K*. *scoparia* selected line across the harvest dates (4 to 11 WAT) (Fig 1).

Similar to vegetative growth parameters, the reproductive parameters of S plants were different from R plants in the absence of competition. The flower initiation occurred on average 25 d earlier in S vs. R selected line (Table 3). The R produced an average of 13,982 seeds plant^-1^ compared with the 23,032 seeds plant^-1^ produced by the S *K*. *scoparia* selected line (P = 0.006). The R had a 1000-seed mass of 1.6 g, which was 38% less than the S *K*. *scoparia*; R seeds were smaller in size compared with the S *K*. *scoparia* seeds.

**Table 3:** Reproductive parameters of dicamba-fluroxypyr–susceptible (S) and –resistant (R) *K*. *scoparia* from the noncompetitive growth study averaged over experimental runs (N = 12).

Selected line^a^	Time to flower initiation (no. of days)^b^	Seed yield (no. plant^-1^)	1000-seed mass (g)
**S**	81 b	23,032 a	2.6 a
**R**	106 a	13,982 b	1.6 b

^a^ S, dicamba-fluroxypyr–susceptible selected line; R, dicamba-fluroxypyr–resistant selected line.

^b^ For each parameter, means within a column followed by the same letter are not significantly different based on the Fisher’s protected LSD test (α = 0.05).

### Competitive growth

**Fig 2:**
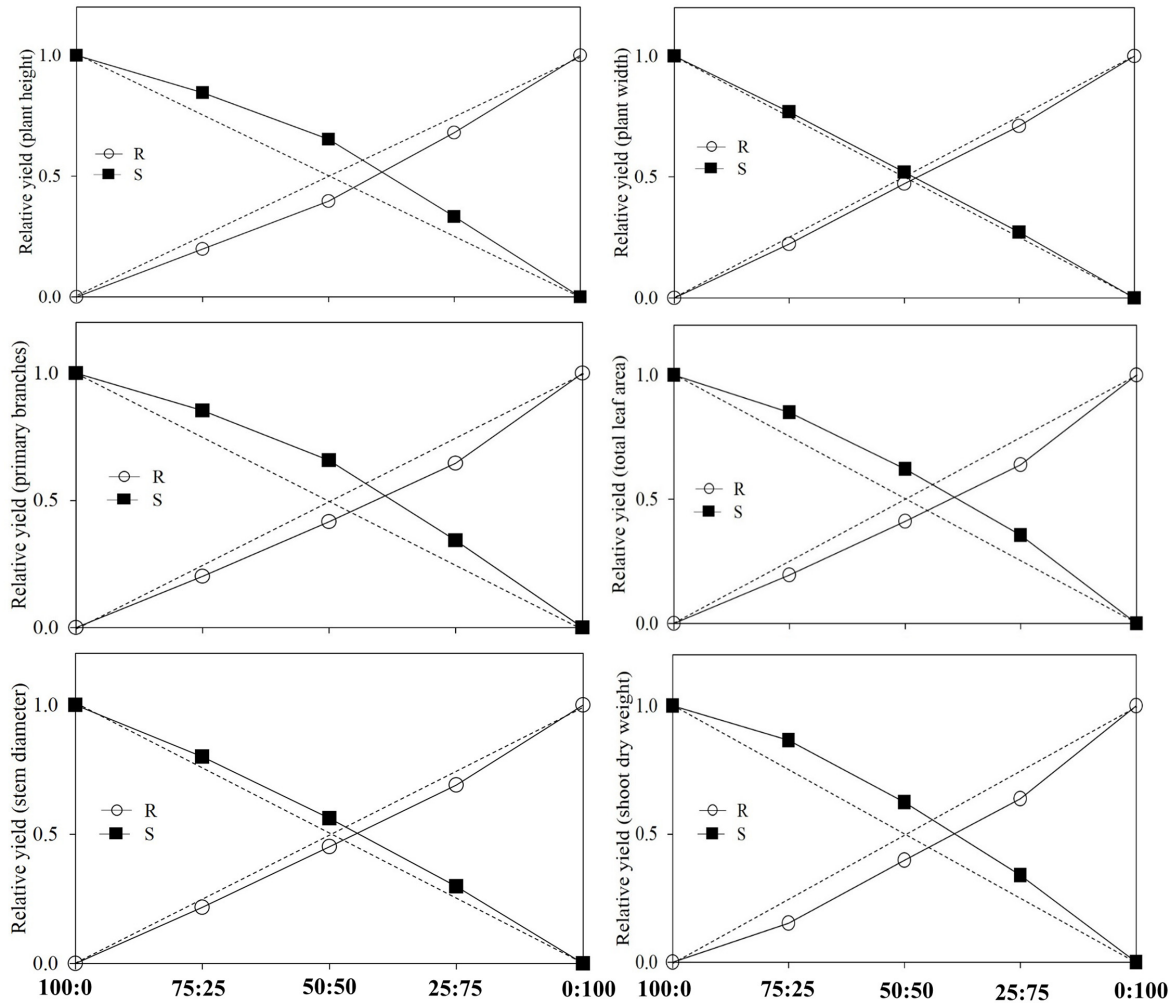
Estimated relative yield (RY) for vegetative growth parameters under different mixture proportions of dicamba-fluroxypyr–susceptible (S) and –resistant (R) *K*. *scoparia* in the replacement series experiments averaged over experimental runs. Dashed lines indicate the expected hypothetical values (H_0_) for RY when the two selected lines are equally competitive.

The relative fitness of S vs. R *K*. *scoparia* selected lines was assessed by the estimated indices of the vegetative growth parameters (plant height, plant width, primary branches, total leaf area, stem diameter, shoot dry weight) obtained from the replacement series experiments. If two weed biotypes grown at different mixture proportions are equally competitive, it is expected that the value of RY index will be 0.25, 0.50, and 0.75 for 25:75, 50:50, and 75:25 mixtures, respectively [33–35]. However, if RY index deviates significantly from the expected value (H_0_), it can be concluded that the two biotypes differ in their competitive ability and demand for resources when grown in competition [33, 35]. The calculated values of RY for S and R differed significantly from H_0_ values for a majority of the vegetative growth parameters (Fig 2). Furthermore, for plant height, primary branches, total leaf area, stem diameter, and shoot dry weight, the RY index values of the S selected line (in mixture proportions of 75:25, 50:50, 25:75) were significantly greater than the corresponding H_0_ values; whereas, R had significantly lower RY index values than the corresponding H_0_ values (Fig 2). These results indicate that S and R plants when grown in intraspecific competition will differ in their demand for resources [33–35], and the S was relatively more competitive than the R *K*. *scoparia*.

Other replacement series indices (CR, RCC, and AI) also helped in determining the aggression levels between the two *K*. *scoparia* selected lines. It is expected that one biotype (1) will be more aggressive than the other biotype (2) or vice versa, if the estimated values of CR > 1.0, RCC (1) > RCC (2), and AI > 0 [33, 35]. Likewise, the biotypes under comparison are considered equally competitive, if CR = 1, RCC (1) = RCC (2), and AI = 0 [33, 35]. Our results indicated that the estimated values of CR and AI indices were significantly different from the expected values of 1.0 and 0, respectively, and values for RCC (S) were significantly different from RCC (R) for all the observed vegetative growth parameters, except stem diameter (Table 4). These results further demonstrate that the S was relatively more competitive than the R *K*. *scoparia*.

**Table 4:** Estimated indices for growth parameters of dicamba-fluroxypyr–susceptible (S) and –resistant (R) *K*. *scoparia* in the replacement series experiments averaged over experimental runs.

Parameters	Indices (S vs. R)^a^^,^ ^b^^,^ ^c^						
	CR	P value^d^	RCC (S)	RCC (R)	P value^e^	AI	P value^f^
**Plant height**	1.648	0.008	1.879	0.656	0.003	0.257	0.004
**Plant width**	1.099	0.001	1.083	0.898	0.005	0.047	0.002
**Primary branches**	1.576	0.002	1.914	0.716	0.001	0.240	0.003
**Total leaf area**	1.511	0.004	1.643	0.699	0.005	0.210	0.003
**Stem diameter**	1.007	0.871	1.046	1.033	0.891	0.003	0.902
**Shoot dry weight**	1.567	0.001	1.660	0.662	0.002	0.226	0.006

^a^ S, dicamba-fluroxypyr–susceptible selected line; R, dicamba-fluroxypyr–resistant selected line.

^b^ CR is the competitive ratio; RCC (S) and RCC (R) are the relative crowding coefficients of S and R selected lines, respectively; and AI is the aggressiveness index.

^c^ Indices were estimated only for equal mixture proportions (50:50) of S and R *K*. *scoparia*.

^d^ P values for one-sample *t* test for determining the deviation of CR from 1.0 (α = 0.05).

^e^ P values for student’s *t* test for the comparison of RCC (S) and RCC (R) (α = 0.05).

^f^ P values for one-sample *t* test for determining the deviation of AI from 0 (α = 0.05).

## Discussion and Conclusions

Delayed germination of the R line (as evident from either I_50_ or I_90_ values) at low constant temperatures of 5, 10 and 15°C and at low alternating temperatures of 5/10 and 10/15°C indicate that the R line exhibits a higher thermal requirement for germination than the S line. In the Northern Great Plains (NGP), the average soil temperature does not exceed 15°C in the spring (March/April) and ranges between 30 and 35°C during mid to late summer (June/July). Based on the results, the R individuals are more likely to escape pre-sowing weed control practices during early spring in Montana and other states of the NGP. This escape mechanism by delaying germination has previously been documented in *K*. *scoparia* populations from maize fields in Nebraska, where the herbicide isoxaflutole was in continuous use to control *K*. *scoparia* for > 8 years [30]. Similarly, a relatively lower proportion of the resistant *K*. *scoparia* population is expected to emerge late in the season in the NGP, evident from the delayed germination of the R vs. S, at least at constant temperatures of 30 and 35°C. Overall, the dicamba-fluroxypyr–resistant population is likely to be more dormant, and potentially more persistent in the soil seed bank, than the S population in the absence of dicamba-fluroxypyr. However, besides dormancy, longevity of the seed bank also requires defenses against seed mortality factors, including pre-plant tillage or preemergence herbicide, pathogen attack, ageing, and predation by herbivores, birds, or insects. The defense against these factors might be especially important because *K*. *scoparia* seeds are short-lived in the soil [2].

Pleiotropic effects associated with mutations endowing herbicide resistance play a significant role in the evolution of adaptive traits in arable weeds [36]. This includes changes in germination dynamics and seedling emergence, also reported in the grass weed *Alopecurus myrosuroides* L., that have mutations in acetyl-coenzyme A carboxylase (ACCase) alleles conferring resistance to ACCase-inhibitor herbicides [36]. A delayed germination due to the pleiotropic effect of resistance alleles has also been reported in ACCase-resistant *Lolium rigidum* Gaud. [37].

The differences in vegetative growth parameters including plant height, plant width, primary branches, total leaf area, stem diameter, and shoot dry weight plant^-1^ between R and S *K*. *scoparia* selected lines clearly indicated that the S plants were more competitive than the R plants in the absence of competition. This may be attributed to physiological differences, resulting in a more efficient utilization of the resources available to each plant for dry matter production, in the S vs. the R line [24]. Also in a growth chamber study, it was found that the auxinic-resistant (cross resistance to dicamba, 2,4-D, picloram, MCPA) *S*. *arvensis* plants produced less shoot dry weight relative to the susceptible plants [21].

Additionally, the R *K*. *scoparia* selected line produced less seeds plant^-1^ compared to the S selected line, suggesting a fitness disadvantage. Fecundity is an ultimate measure of the fitness cost associated with herbicide resistance alleles [23, 25]. A dicamba/2,4-D-resistant biotype of *Brassica kaber* L. also produced less seeds than the susceptible biotype (3,120 vs. 2,520 seeds plant^-1^). Similarly, the auxinic-herbicide–resistant plants of *S*. *arvensis* produced less seeds (10 seeds plant^-1^) compared with the auxinic-herbicide–susceptible plants (25 seeds plant^-1^) [21]. Also, an MCPA-resistant *R*. *acris* biotype was less competitive and had lower seed yield than the susceptible biotype [24]. The lower 1000-seed mass further indicated that the dicamba-fluroxypyr–resistant *K*. *scoparia* individuals would have a lower rate of survivorship at the seedling phase of development compared with the S individuals. Seed mass or seed size is the indicator of progeny fitness, including radicle and coleoptile growth, early seedling growth, and plant biomass [38]. The lower competitive ability and aggressiveness of the R relative to the S *K*. *scoparia* selected line was also evident when grown at different mixture proportions in the replacement series experiments. One phenotype with a higher RCC in the mixture is expected to have a competitive advantage and a higher survivorship over the other phenotype [24, 33].

The relative fitness (w) of an HR biotype, calculated as the seed production of the HR biotype/seed production of the susceptible biotype [22, 23], was 0.61 for the R relative to the S *K*. *scoparia*, which means that the resistant plants will exhibit a 39% fitness cost (1-w) in the absence of the auxinic herbicides [22, 23]. This implies that the relative frequency of dicamba-fluroxypyr–resistant individuals is likely to decline in the population when the use of auxinic herbicides is discontinued [39].

This study does not distinguish between the processes of survival, fecundity, and competition to ascertain the ecological fitness of the R relative to the S selected line. However, it does highlight relative differences in the germination dynamics in response to thermal environment and in competitive ability and yield per plant in the absence of competition or at a density of 84 plants m^-2^ between the two contrasting *K*. *scoparia* selected lines. These phenotypic changes most likely associated with the pleiotropic effects of the auxinic-herbicide resistance alleles, can confer a substantial fitness variation during the *K*. *scoparia* life cycle [16, 23, 24, 25], which will have practical implications. A fitness cost associated with resistance to auxinic herbicides may explain the slow evolution and spread of auxinic-herbicide–resistant weed biotypes, despite the extensive use of auxinic herbicides for more than 60 yr [14, 16, 21, 25, 26]. Future research needs understanding the mechanism of differential fitness between the R and S individuals. This study also emphasizes the need to elucidate the underlying mechanism(s) of resistance in the selected dicamba-fluroxypyr–resistant *K*. *scoparia*.

Evident from this research, the competitive penalties associated with the R vs. the S selected line, is novel information that will aid in developing simulation models to predict the evolutionary dynamics of auxinic-herbicide resistance in *K*. *scoparia* populations. These findings are also critical in the context of recent development and commercialization of dicamba-tolerant crops as a tool to manage glyphosate-resistant weeds. It becomes even more critical when glyphosate, ALS, and triazine-resistant *K*. *scoparia* populations are widespread in this geography [10]. The adoption of this new herbicide-tolerance trait technology will possibly increase the use of dicamba, resulting in a higher selection pressure for resistance development, if not proactively managed. Moreover, there were no differences in fitness traits of glyphosate- and ALS-inhibitor-resistant compared to -susceptible biotypes of *K*. *scoparia* [7, 40]. Given this scenario, growers should follow the dicamba-use stewardship in crop or chemical fallow, and carefully monitor the response of *K*. *scoparia* populations to dicamba and fluroxypyr applications in their production fields. Compared to other HR weeds, best management practices (BMPs) for dicamba-fluroxypyr R *K*. *scoparia* might be different because of the competitive penalties. Therefore, if the selection pressure from dicamba or fluroxypyr is interrupted by other weed control methods such as effective, alternative site-of-action PRE/POST herbicides, tillage, mowing, late-season herbicides, or by other BMPs [6, 41, 42, 43, 44], the proportion of R to S in the soil seedbank will decrease. This will ultimately cause a decline in the R population of *K*. *scoparia* in the field.

Finally, ensuring a common genetic background of R and S individuals is a fundamental requirement for conducting fitness-cost studies [22, 23]. The numerous, small inconspicuous flowers borne in the leaf axil of the entire *K*. *scoparia* plant makes crossing efforts extremely difficult and time consuming [2, 7, 8]. Although we selected R and S individuals from within a single field-collected population through recurrent group selection [45–48], the assumption of a common genetic background needs further evaluation, especially for a protogynous, outcrossing species like *K*. *scoparia*. The issue of genetic purity is further complicated by the tumbling mechanism of seed dispersal that can distribute seeds across large distances [2, 5, 49]. Therefore, to unequivocally attribute fitness cost endowed by the auxinic herbicide-resistance alleles, and rule out the possibility of ecotypic differentiation, the results from this study needs further validation by creating near-isogenic lines of R and S or segregating F_2_ populations [22, 23, 50]. Alternatively, the fitness-cost study can be conducted under field conditions using several, at least six, each of R and S *K*. *scoparia* populations, which will aid in minimizing the effect of differences in the genetic background [22, 23].

## Supporting Information

S1 Dataset Germination at Constant and Alternating temperatures. (XLSX)

S2 Dataset Noncompetitive Growth Study. (XLSX)

S3 Dataset Competitive Growth Study. (XLSX)
